# Pim kinases in hematological malignancies: where are we now and where are we going?

**DOI:** 10.1186/s13045-014-0095-z

**Published:** 2014-12-10

**Authors:** Patrizia Mondello, Salvatore Cuzzocrea, Michael Mian

**Affiliations:** Department of Human Pathology, University of Messina, Via Consolare Valeria, Messina, 98125 Italy; Department of Biological and Environmental Sciences, University of Messina, Messina, Italy; Department of Hematology, Hospital S. Maurizio, Bolzano/Bozen, Italy; Department of Internal Medicine V, Hematology & Oncology, Medical University Innsbruck, Innsbruck, Austria

**Keywords:** Pim kinases, Target therapy, Hematologic malignancies, Pim inhibitors

## Abstract

The proviral insertion in murine (PIM) lymphoma proteins are a serine/threonine kinase family composed of three isoformes: Pim-1, Pim-2 and Pim-3. They play a critical role in the control of cell proliferation, survival, homing and migration. Recently, overexpression of Pim kinases has been reported in human tumors, mainly in hematologic malignancies. In vitro and in vivo studies have confirmed their oncogenic potential. Indeed, PIM kinases have shown to be involved in tumorgenesis, to enhance tumor growth and to induce chemo-resistance, which is why they have become an attractive therapeutic target for cancer therapy. Novel molecules inhibiting Pim kinases have been evaluated in preclinical studies, demonstrating to be effective and with a favorable toxicity profile. Given the promising results, some of these compounds are currently under investigation in clinical trials. Herein, we provide an overview of the biological activity of PIM-kinases, their role in hematologic malignancies and future therapeutic opportunities.

**Electronic supplementary material:**

The online version of this article (doi:10.1186/s13045-014-0095-z) contains supplementary material, which is available to authorized users.

## Introduction

The proviral insertion in murine (Pim) lymphoma family proteins, whose gene locus was discovered as a proviral integration site for Moloney murine leukemia virus infection, consists of three serine/threonine kinase isoforms: Pim-1, Pim-2 and Pim-3 [1]. These proto-oncogenic kinases are constitutively active and they are mainly regulated at the transcriptional and translational level [2],[3] by cytokines and growth factors involved in hematopoiesis, such as interleukin (IL)-2, IL-3 [4],[5], IL-6, granulocyte-macrophage colony-stimulating factor (GM-CSF) and granulocyte-colony stimulating factor (G-CSF) [6]. Furthermore, the stability and function of Pim kinases depend on their interaction with heat shock protein (Hsp) 90, a chaperone protein involved in folding and stabilizing different molecules [7]. Hsp90 showed not only to protect Pim-1 from ubiquitin-26S-proteasomal degradation, but also to mainten the proper conformation of Pim-1 [8].

Pim kinases play a critical role in the control of cell proliferation and survival. They are downstream effectors of important oncoproteins, such as Ableson (ABL) [9], Janus Kinase 2 (JAK2) [10] and FMS-like tyrosine kinase 3 (FLT3) [11]. Although Pim kinases exert similar functions, they have different tissue distributions [12],[13]. While Pim-1 and Pim-2 are predominantly expressed in hematopoietic cells [12],[14], Pim-3 expression is high in brain, kidney, and epithelia [12],[15]. Due to their aberrant expression in human tumors [16]-[19], they could be important contributors in the pathogenesis of neoplasias including lymphomas, gastric, colorectal and prostate cancers [20].

The oncogenic potential of Pim kinases has been studied on transgenic mouse models. In the Eμ-pim1 model only 5–10% of mice developed T-cell lymphoma, suggesting that Pim-1 alone is not able to induce a massive proliferation [21]. Interestingly, infection of these transgenic mice with murine leukemia virus (MuLV) promoting the integration of the provirus in the Pim-1 locus [22] enhanced dramatically the incidence of tumors and reduced the latency of T-cell lymphoma development [21]. The activation of either c-Myc or N-Myc was involved in every tumor, suggesting an oncogenic collaboration between Myc and Pim-1 genes in lymphomagenesis [21],[23]. Co-expression of both Eμ–Pim1 and Eμ–Myc was incompatible with life, leading the transgenic mice to succumb to lymphomas *in utero* or around birth. Conversaly, Eμ-Myc;Eμ-Pim1 mice with low expression of c-Myc were viable and with low tumor incidence [24].

The oncogenic role of Pim-1 and its cooperation with c-Myc have also been studied in prostatic cancer. Pim-1 demonstrated to promote prostate tumorgenesis by enhancing the transcriptional activity of androgen receptors. Notably, Pim1-expressing cells presented an increased c-Myc transcriptional activity as well. Treatment with the c-Myc inhibitor 10058-F4 reduced Pim-1 protein and suppressed the tumorigenicity of the prostate cancer cells [25]. In addition, Pim kinases have been demonstrated to cooperate with other oncogenes, such as bcl2 [26], bcl6 [27], runx2 [28], E2a-pbx1 [29], frat1 [30].

PIM knock-out studies have shown that mice deficient in all three Pim kinases are viable and fertile, supporting the tolerability of pan-Pim inhibition [12]. Mikkers et al. demonstrated that the lack of these kinases resulted in only a decrease of erythrocyte mean cell volume (MCV) [12]. However, a recent study has proved that the triple PIM knock-out affected multiple lineages of hematopoietic cells as well as the self-renewal of hematopoietic stem cells (HSCs) [31]. Based on these results a careful monitoring of potential hematological side effects is recommended with the Pim inhibitors treatment.

In this review we provide an overview of the biological background of Pim kinases, their role in hematologic malignancies and a summary of possible drugs targeting theses enzymes.

### The oncogenic potential of PIM kinases

**Figure 1:**
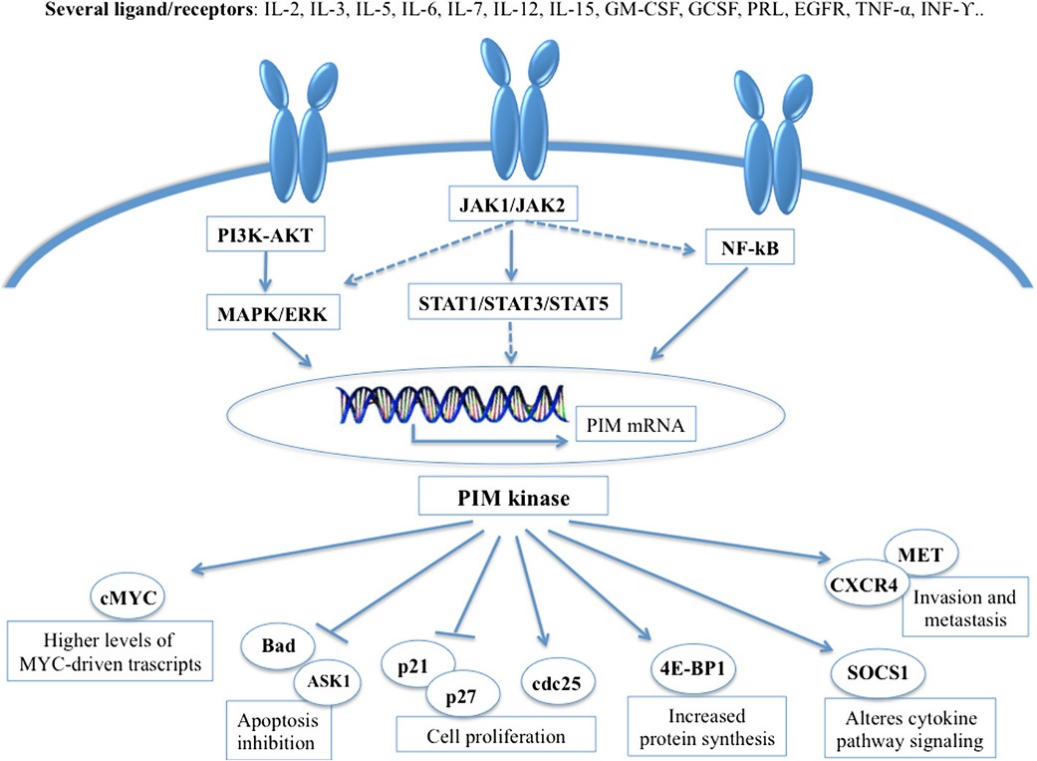
**Mechanisms regulating Pim levels and downstream activation.**

Pim kinases are critical components of distinct pathways that play an important role in cell proliferation and survival [32]-[34] (Figure 1) and especially in apoptosis, cell cycle regulation, cell proliferation and cell migration.

#### Apoptosis

Pim kinases prevent cells from apoptosis by phosphorylating the proapoptotic Bcl-2–associated agonist of cell death (Bad). Phosphorylation of Bad on Serine (Ser) 112 and Ser136, respectively by Pim-1 and Pim-2, induces 14-3-3 binding, which results in loss of the binding with the anti-apoptotic protein Bcl-2 and, consequently, in cell survival [35]-[37]. Similarly, phosphorylation of Bad on Ser155 by Pim-3 was found to prevent Bad from binding to the anti-apoptotic protein Bcl-xL [38]. In addition, the pro-survival activity of Pim kinases seems to depend also on direct phosphorylation of the apoptosis signaling kinase 1 (ASK1) [39], which decreases significantly ASK1 activity and inhibits ASK1-mediated phosphorylation of JNK and p38. Ultimately this phosphorylation event leads to blocking caspase-3 activation and decreasing apoptosis levels [39].

Pim kinases usually phosphorylate Mdm2 on Ser166 and 186, an E3 ubiquitin ligase which mediates ubiquitylation and proteasome-dependent degradation of p53 [40]. Notably, these residues are targets of other signaling pathways including Akt [41]-[46]. When Pim kinases are overexpressed, such as in tumors, they block the degradation of both p53 and Mdm2 in a Mdm2-independent manner, leading to an increase of p53. In addition, Pim-1 enhances p14ARF activity [40], a Mdm2 inhibitor well known to arrest the degradation of both p53 and Mdm2 itself [47],[48].

Finally, Pim-2 maintains high levels of NFkB required for its antiapoptotic function. Indeed, transcriptional targets of the NF-kB include many genes associated with survival, such as Bcl-2 and Bcl-xL. Hammerman et al. demonstrated that Pim-2 activates NF-kB by inducing phosphorylation of Cot, a serine/threonine kinase downstream to both MAPK/ERK and NF-kB signaling pathways [49]. Furthermore, Pim-1 phosphorylates RelA/p65, the main subunit of NF-kB, preventing its degradation from ubiquitin-mediated proteolysis. Knocking down Pim-1 severely impaired cell survival, at least in part, by interfering RelA/p65 activation [50].

#### Cell cycle regulation

Pim kinases are involved in cell proliferation through the phosphorylation of the cyclin-dependent kinase inhibitors p21 at Threonine (Thr)145 and Ser146 [51],[52], p27 at Thr157 and Thr198 [53]. Phosphorylation of p21 induces its translocation from the nucleus to the cytoplasm, resulting in cell proliferation and survival [54],[55]. Overexpression of Pim-2 leads to enhanced levels and stability of p21, while knockdown of Pim-2 results in reduced levels of p21 [52]. Notably, treatment with pan-Pim inhibitor lowered not only Pim-2 kinase activity, but also p21 phosphorylation [56]. An inverse relation seems to exist between Pim and p27 levels. Morishita et al. have demonstrated that phosphorylation of p27 by Pim kinases prompts its binding to 14–3–3 proteins and subsequent nuclear exclusion and degradation of p27. Furthermore, Pim kinases seem to down-regulate p27 at the transcriptional level by inactivating Forkhead transcription factors [53]. In addition, Pim-1 seems to influence cell cycle phase transition by phosphorylating critical tyrosine phosphatases: Cdc25A for the G1/S and Cdc25C, and the Cdc25C-associated kinase (C-TAK)1 for the G2/M [57],[58].

In further support of its role in the cell cycle, Pim-1 showed to phosphorylate the nuclear mitotic apparatus protein (NuMa), that is essential for mitotic spindle formation and aids in chromosome separation. Pim-1 seems to contribute to maintenance of a stable connection between NuMA, dynein/dynactin and the pericentric protein HP1β, a complex that is necessary for mitosis. Notably, the ‘kinase-dead’ mut-Pim-1-GFP fusion protein hinders the progression of mitosis and results in rapid cell death by apoptosis [59].

#### Cell Proliferation

Pim-1 and/or Pim-2 are significant downstream targets of transcription factors STAT 3 and STAT 5 [60]. Pim kinases in turn are able to influence the JAK/STAT pathway through their direct interaction and phosphorylation of Suppressor of Cytokine Signalling (SOCS)-1, a well-known regulator of this signaling pathway [61],[62]. The modulation of SOCS1 by Pim kinases seems to have a critical role in v-Abl-induced tumorgenesis. In the xenograft model v-Abl was not able to transform bone marrow cells deficient in the Pim-1/Pim-2 model, but it partially succeeded in its transformation activity combining the triple knockout of Pim-1, Pim-2 and SOCS1 [63]. Although Pim kinases are pro-oncogenic proteins, they are not sufficient to initiate disease [64] and therefore a cooperation between Pim-1 and c-Myc is required for promoting STAT3-mediated cell cycle progression [65]. Both Pim-1 and Pim-2 phosphorylate c-Myc, increasing its stability and consequently its transcriptional activity [66]. Recently, it has been demonstrated that this cooperation takes place also at the transcriptional level through Pim-mediated phosphorylation of preacetylated hystone H3 on Ser10 (H3S10) [67]. The H3S10 phospholytation is required to mediate the interaction with 14-3-3 proteins [68] and the ensuing recruitment of the histone acetyltransferase MOF, which acetylates histone H4, generating a nucleosome platform for bromodomain-containing protein 4 (BRD4) binding. Thereafter, the positive transcription elongation factor b (P-TEFb) is recruited inducing the phosphorylation of RNA polymerase II (Pol II) and the release of stalled Pol II, which activates transcriptional elongation [69]. However, Pim kinases contribute for about 20% of Myc-induced gene expression [67]. c-Myc stability is mainly controlled by the ubiquitin–proteasome system, in particular by Fbxw-7 [70]. An inverse proportion exists between Fbxw-7 and c-Myc levels [71],[72], consequently Fbxw-7 deletion was associated with c-Myc accumulation and aberrant cell cycle entry [73],[74]. Mutation of Fbxw-7 has been found in solid [75]-[78] and blood malignancies [79]. In addition, also the control of miRNA expression plays an important role in Myc-mediated tumorgenesis. Several studies found that c-Myc repressed a wide range of miRNAs by directly binding the promoter of these miRNAs [80],[81], favoring Myc-mediated tumorgenesis and conferring a more aggressive feature.

Pim activity is synergistic with another independent pro-survival pathway, phosphoinositide-3 Kinase/Akt/mammalian (PI3K/Akt/mTOR) [82]. Pim-2, but not Pim-1, has been identified as the principal kinase that phosphorylates the translational repressor 4E-BP1 and p70S6 independently of the PI3K/Akt/mTOR pathway [2]. Indeed, the activation of Pim-2 revealed an important role in cell growth resistant to rapamicin, an mTOR inhibitor [83]. Therefore, suppressing Pim-2 is important to treat rapamycin-resistant tumors.

#### Cell migration

The Pim proteins also revealed an involvement in signaling pathways that control cell migration. Pim-1 plays a significant role on MET expression, the receptor for hepatocyte growth factor (HGF) involved in signaling normal and tumor cell migration and invasion. Pim-1 controls the translation of MET by phosphorylation of eukaryotic initiation factor 4B (eIF4B) at Ser406 and the use of Pim inhibitor has been associated with a significant decrease of MET expression [84].

In addition, Pim-1 showed an influence on the chemokine ligand 12 (CXCL12)/chemokine (C-X-C motif) receptor 4 (CXCR4) expression, a ligand/receptor system with a crucial role in normal homeostasis [85], especially in the hematopoietic stem cells grafting [86],[87]. Extracellular signal-regulated protein kinase 1/2 (ERK1/2) [88],[89], PI3K [90] and Syk [91] transduction pathways showed to be differently implicated in CXCR4-mediated migration and proliferation. The imbalance of CXCL12/CXCR4 axis is implicated in cancer progression and spreading of tumor-initiating cells [92]. In vitro experiments suggested that Pim-1 might regulate CXCR-4 expression through phosphorylation of the Ser339 in the intracellular domain involved in receptor internalization [93]. Pim-1 overexpression has proved to correlate with CXCR4 levels also in leukemic blasts. Interestingly Pim inhibitor treatment led to downregulation of CXCR4 surface expression in primary cells, inducing impairment of Pim-mediated cell survival and block of CXCR4-mediated interaction of cells with their protective microenvironment [93],[94].

### Expression levels of PIM kinases in hematologic cancer

Overexpression of Pim kinases has been observed in different human cancers, but mainly in haematological malignancies [20]. Initially, overexpression of Pim-1 was found in human myeloid and lymphoid leukemias [33],[34]. In acute myeloid leukemia (AML) increased levels of Pim-1 have been associated with aberrant expression of the mixed-line-age leukemia (MLL) gene [95] as a consequent activation of tyrosine-kinase receptor FLT3 or the transcriptional regulator Hoxa9 [11],[96]-[98].

Almost half of diffuse large B-cell lymphoma (DLBCL) tissues showed an increased expression of Pim kinases [99],[83], which is even more frequent in the activated B-cell (ABC) subtype due to the constitutive activation of JAK/STAT3 signaling [14]. Brault et al. proved a strong correlation between level expression of Pim kinases, STAT signaling, higher proliferative rate, and more advanced disease stage. Therefore, these findings suggest the possible role of Pim kinases as markers for DLBCL progression [100].

Increased levels of Pim-2 were also found in mantle cell lymphoma (MCL), follicular lymphoma (FL), marginal zone lymphoma-MALT type (MZL-MALT), chronic lymphocytic leukemia (CLL), nodal marginal zone lymphoma (NMZL) [17],[101],[102] and multiple myeloma [103].

### Pim kinases as therapeutic targets

Pim kinases are attractive therapeutic targets since they are often aberrantly expressed in several hematologic disorders and because they contribute to cellular proliferation and migration. A large number of new molecules have been produced so far. While most of them are specific inhibitors of Pim-1, only few are able to inhibit all Pim isoforms [104],[105] (Table 1). However, because of the overlapping functions of these kinases, a pan-Pim inhibitor resulted to be more effective than a selective one [106].

**Table 1:** **Novel Pim-Inhibitors in hematologic malignancies**

Compound	Class	PIM inhibition selectivity	Development	Disease
**SGI-1776**	imadizaopyridazine	IC50: 7 nM PIM1, 363 nM PIM2, 69 nM PIM3, 44 nM FLT-3 and 34 nM Haspin [107].	Failure in phase I clinical trials by cardiotoxicity	Non-Hodgkin lymphoma
**SMI4a**	benzylidene-thiazolidene-2,4-dione	IC50: 21 nM PIM1, 100 nM PIM2 [113].	Preclinical	Acute myeloid leukemia
		Selective vs. 56 kinases [114].		
**LGB321**	3-(S)-amino-piperidine pyridyl carboxamide	IC50: 0.001 nM PIM1, 0.0021 nM PIM2, and 0.0008 nM PIM3 [106].	Recruiting patients for clinical trials	Multiple myeloma
**AZD1897**		IC50: 3 nM PIM1,2 and 3 [115].	Preclinical	Acute myeloid leukemia
**SEL24-B58**	Benzoimidazol	IC50: 31 nM PIM1, 154 nM PIM2, 152 nM PIM3. Selective in a panel of 299 kinases with the exception of haspin, HIPK and CLK kinases [117].	Preclinical	Leukemic monocyte lymphoma
**AZD1208**	thiazolidene	IC50: Pim-1 0.4 nM, Pim-2 5.0 nM and Pim-3 1.9 nM [115].	Recruiting patients for clinical trials	Acute myeloid leukemia

A first generation of Pim-inhibitors (SGI-1773, SGI-1776) demonstrated high antitumor activity both in vitro and in vivo in different hematological tumors [107]-[109]. SGI-1776 is an imadizaopyridazine with nanomolar activity not only on the three Pim kinases, but also on Flt3 and Haspin. Therefore, the real contribution of Pim inhibition to the efficacy of this compound is unclear. In contrast to SGI-1773, SGI-1776 has shown to induce an almost complete suppression of Cyclin D1, cMYC and MCL1. In addition, SGI-1776 has been demonstrated to overcome Pim2-mediated rapamycin resistance without increased toxicities in a mouse model [110]. Based on these positive results, a phase I clinical trial recruiting castration-resistant prostate cancer or relapsed/refractory non-Hodgkin lymphoma patients was conducted. However, due to unexpected severe cardiotoxicity the trial was ended early. (NCT00848601) This event could be related to the inhibition of Pim-1, which has been demonstrated to play an important role in the promotion of cardioprotective signaling and inhibiting pathological injury [111],[112].

Second generation Pim-inhibitors were designed to increase specificity and to eliminate cardiotoxicity. SMI4a is a benzylidene-thiazolidene-2,4-dione inhibiting PIM1 (24 nM) and PIM2 (100 nM) [113]. This molecule is able to induce G1 arrest through a marked increase of p27 and consequently inhibition of cdk2. In a mouse model SMI4a induced a delay in tumor growth without important toxicity [113]. SMI4a demonstrated synergy with the mTOR inhibitor rapamycin by downregulating p4EBP1 and blocking proliferation in AML cells [114]. Since SMI4a increases phosphorylation of ERK1/2, its association with a MEK1/2 inhibitor also showed a good synergistic activity leading to a higher death rate of precursor T-cell lymphoblastic lymphoma cells [113].

LGB321 is a 3-(S)-amino-piperidine pyridyl carboxamide, ATP-competitive inhibitor of all three Pim kinases with a 50% inhibitory concentration (IC50) for Pim-1, Pim-2 and Pim-3 of 0.001, 0.002 and 0.0008 nM, respectively. LGB321 was tested in different hematologic cell lines such as ALL, AML, multiple myeloma and B-cell NHL. Among all studied cell lines, the multiple myeloma subtype was the most sensitive with IC50 values in the picomolar range. LGB321 efficacy and safety profiles were also confirmed in vivo models [106]. Based on these data, a phase I clinical trial evaluating the LGB321 activity in relapse/refractory myeloma is ongoing (NCT02144038).

AZD1897 is an ATP-competitive pan-Pim inhibitor with IC50 of 3 nM against Pim-1, 2 and 3 [115], recently evaluated in treatment of AML. In vitro studies demonstrated a limited activity of AZD1897 as a single agent, but a strong synergy in combination with the AKT-inhibitor AZD5363. This association led to a greater cytotoxic activity as well as a decreased downstream mTOR-targets (p4EBP1, pS6 kinase) and MCL1 levels with respect to the single agent therapy [116]. Based on the remarkable anti-leukemic activity of AZD1897 combined with AKT inhibition future clinical trials are warranted.

SEL24-B58 is able to inhibit all three Pim kinases already at picomolar dose (IC50 values are: Pim-1 31 nM, Pim-2 154 nM and Pim-3 152 nM). In vitro SEL24-B58 reduced Mcl-1 levels, demonstrating synergy in combination with the Bcl2-family inhibitor ABT-737 in leukemic monocyte cells. The combination with JAK1/2 inhibitor (Cyt387) in lymphoblastic leukemia cells resulted in a synergistic antiproliferative activity as well. A Xenograft model confirmed the efficacy of SEL24-B58 at a concentration of 150 mg/kg with a completed arrest of tumor growth after 17 days of treatment and no toxicity [117].

AZD1208 is a thiazolidene, highly selective for Pim-1, Pim-2 and Pim3 with a low nanomolar activity in cells (IC50 of 0.4 nM for Pim-1, 5.0 nM for Pim-2, and 1.9 nM for Pim-3) [115]. AZD1208 demonstrated in vitro and in vivo activity against AML. A significant growth inhibition was evident in a dose-dependent manner. Indeed, AZD1208 led to decreased phosphorylation of Bad, 4EBP1, p70S6K, and S6, as well as increased cleaved caspase 3 and p27 [32]. Notably, it showed to be active in Flt3-ITD primary tumor cells without the off-target inhibition activity [118] unlike previous PIM inhibitors [108],[109],[119]. Based on these interesting data, AZD1208 is currently being evaluated in phase 1 clinical trials. (NCT01489722, NCT01588548).

## Conclusion

Pim kinases create a wide interest in oncology due to their overexpression in cancer and association with enhanced tumor growth and chemo-resistance. Given the close advent of Pim inhibitors in clinic, it is important to find their most efficient application. First of all biomarker identification might allow to select the patients and follow the course of treatment. So far, no genetic markers have been established to guide therapeutical decision. Therefore, it may be worth improving the knowledge of Pim-dependent gene expression and verify the existence of a correlation between high levels of different subsets of Pim-regulated genes and increased sensitivity to treatment. Another open question is whether Pim kinase inhibitors should be used as monotherapy or in combination. Preclinical data have shown that Pim inhibitors are effective drugs when used as single agents. However, their positive effect was even more pronounced when they were combined with chemo- or other target-therapies (Pi3k/AKT/mTOR inhibitor). In addition, these inhibitors demonstrated to significantly reverse drug resistant phenotypes in preclinical models. It is necessary to wait until the conclusion of clinical trials using Pim kinases inhibitors to see if tumor cells will develop resistance through other signaling pathways. Finally, evaluation of toxicity will be important as well due to the difficulty in finding the right balance between sufficient inhibition and acceptable toxicity when multiple signaling inhibitors are combined.

In the near future research should focus on the activity of Pim kinases and their involvement in resistance mechanisms in order to allow for a more efficient treatment and application.

## Authors’ original submitted files for images

Below are the links to the authors’ original submitted files for images. Authors’ original file for figure 1 Authors’ original file for figure 2

## Abbreviations

PIM: Proviral insertion in murine
IL: Interleukin
GM-CSF: Granulocyte-macrophage colony-stimulating factor
G-CSF: Granulocyte-colony stimulating factor
ABL: Ableson
JAK2: Janus Kinase 2
FLT3: FMS-like tyrosine kinase 3
MuLV: Murine leukemia virus
MCV: Mean cell volume
HSCs: Hematopoietic stem cells
Bad: Bcl-2–associated agonist of cell death
Ser: Serine
ASK1: Apoptosis signaling kinase 1
Thr: Threonine
C-TAK: Cdc25C-associated kinase
SOCS: Suppressor of Cytokine Signalling
H3S10: Histone H3 phosphorylate at Ser10
BRD4: Bromodomain-containing protein 4
P-TEFb: Positive transcription elongation factor b
Pol II: Polymerase II
PI3K/Akt/mTOR: Phosphoinositide-3 Kinase/Akt/mammalian
eIF4B: Eukaryotic initiation factor 4B
CXCL12/CXCR4: Chemokine ligand 12/chemokine C-X-C motif receptor 4
ERK1/2: Extracellular signal-regulated protein kinase 1/2
MLL: Mixed-line-age leukemia
ABC: Activated B-cell
MCL: Mantle cell lymphoma
FL: Follicular lymphoma
MZL-MALT: Marginal zone lymphoma-MALT type
CLL: Chronic lymphocytic leukemia
NMZL: Nodal marginal zone lymphoma
IC50: 50% inhibitory concentration

## References

[1] Nawijn MC, Alendar A, Berns A (2011). For better or for worse: the role of Pim oncogenes in tumorigenesis. Nat Rev Cancer.

[2] Fox CJ, Hammerman PS, Cinalli RM, Master SR, Chodosh LA, Thompson CB (2003). The serine/threonine kinase Pim- 2 is a transcriptionally regulated apoptotic inhibitor. Genes Dev.

[3] Qian KC, Wang L, Hickey ER, Studts J, Barringer K, Peng C, Kronkaitis A, Li J, White A, Mische S, Farmer B (2005). Structural basis of constitutive activity and a unique nucleotide binding mode of human Pim-1 kinase. J Biol Chem.

[4] Allen JD, Verhoeven E, Domen J, van der Valk M, Berns A (1997). Pim-2 transgene induces lymphoid tumors, exhibiting potent synergy with c-myc. Oncogene.

[5] Dautry F, Weil D, Yu J, Dautry-Varsat A (1988). Regulation of pim and myb mRNA accumulation by interleukin 2 and interleukin 3 in murine hematopoietic cell lines. J Biol Chem.

[6] Lilly M, Le T, Holland P, Hendrickson SL (1992). Sustained expression of the pim-1 kinase is specifically induced in myeloid cells by cytokines whose receptors are structurally related. Oncogene.

[7] Pratt WB (1997). The role of the hsp90-based chaperone system in signal transduction by nuclear receptors and receptors signaling via MAP kinase. Annu Rev Pharmacol Toxicol.

[8] Mizuno K, Shirogane T, Shinohara A, Iwamatsu A, Hibi M, Hirano T (2001). Regulation of Pim-1 by Hsp90. Biochem Biophys Res Commun.

[9] Nieborowska-Skorska M, Hoser G, Kossev P, Wasik MA, Skorski T (2002). Complementary functions of the antiapoptotic protein A1 and serine/threonine kinase pim-1 in the BCR/ABL-mediated leukemogenesis. Blood.

[10] Wernig G, Gonneville JR, Crowley BJ, Rodrigues MS, Reddy MM, Hudon HE, Walz C, Reiter A, Podar K, Royer Y, Constantinescu SN, Tomasson MH, Griffin JD, Gilliland DG, Sattler M (2008). The Jak2V617F oncogene associated with myeloproliferative diseases requires a functional FERM domain for transformation and for expression of the Myc and Pim proto-oncogenes. Blood.

[11] Kim KT, Baird K, Ahn JY, Meltzer P, Lilly M, Levis M, Small D (2005). Pim-1 is up-regulated by constitutively activated FLT3 and plays a role in FLT3-mediated cell survival. Blood.

[12] Mikkers H, Nawijn M, Allen J, Brouwers C, Verhoeven E, Jonkers J, Berns A (2004). Mice deficient for all PIM kinases display reduced body size and impaired responses to hematopoietic growth factors. Mol Cell Biol.

[13] Eichmann A, Yuan L, Bréant C, Alitalo K, Koskinen PJ (2000). Developmental expression of pim kinases suggests functions also outside of the hematopoietic system. Oncogene.

[14] Bachmann M, Möröy T (2005). The serine/threoninekinasePim-1. Int J Biochem Cell Biol.

[15] Feldman JD, Vician L, Crispino M, Tocco G, Marcheselli VL, Bazan NG, Baudry M, Herschman HR (1998). KID-1, a protein kinase induced by depolarization in brain. J Biol Chem.

[16] Alizadeh AA, Eisen MB, Davis RE, Ma C, Lossos IS, Rosenwald A, Boldrick JC, Sabet H, Tran T, Yu X, Powell JI, Yang L, Marti GE, Moore T, Hudson J, Lu L, Lewis DB, Tibshirani R, Sherlock G, Chan WC, Greiner TC, Weisenburger DD, Armitage JO, Warnke R, Levy R, Wilson W, Grever MR, Byrd JC, Botstein D, Brown PO, Staudt LM (2000). Distinct types of diffuse large B-cell lymphoma identified by gene expression profiling. Nature.

[17] Cohen AM, Grinblat B, Bessler H, Kristt D, Kremer A, Schwartz A, Halperin M, Shalom S, Merkel D, Don J (2004). Increased expression of the hPim-2 gene in human chronic lymphocytic leukemia and non-Hodgkin lymphoma. Leuk Lymphoma.

[18] Wingett D, Long A, Kelleher D, Magnuson NS (1996). Pim-1 proto-oncogene expression in anti-CD3- mediated T-cell activation is associated with pro- tein kinase C activation and is independent of Raf-1. J Immunol.

[19] Cibull TL, Jones TD, Li L, Eble JN, Ann Baldridge L, Malott SR, Luo Y, Cheng L (2006). Overexpression of Pim-1 during progression of prostatic adenocarcinoma. J Clin Pathol.

[20] Shah N, Pang B, Yeoh KG, Thorn S, Chen CS, Lilly MB, Salto-Tellez M (2008). Potential roles for the PIM1 kinase in human cancer - a molecular and therapeuticappraisal. Eur J Cancer.

[21] Van Lohuizen M, Verbeek S, Krimpenfort P, Domen J, Saris C, Radaszkiewicz T, Berns A (1989). Predisposition to lymphomagenesis in pim-1 transgenic mice: cooperation with c-myc and N-myc in murine leukemia virus-induced tumors. Cell.

[22] van Lohuizen M, Verbeek S, Scheijen B, Wientjens E, van der Gulden H, Berns A (1991). Identification of cooperating oncogenes in E mu-myc transgenic mice by provirus tagging. Cell.

[23] Berns A (1991). Tumorigenesis in transgenic mice: identification and characterization of synergizing oncogenes. J Cell Biochem.

[24] Verbeek S, van Lohuizen M, van der Valk M, Domen J, Kraal G, Berns A (1991). Mice bearing the E mu-myc and E mu-pim-1 transgenes develop pre-B-cell leukemia prenatally. Mol Cell Biol.

[25] Kim J, Roh M, Abdulkadir SA (2010). Pim1 promotes human prostate cancer cell tumorigenicity and c-MYC transcriptional activity. BMC Cancer.

[26] Shinto Y, Morimoto M, Katsumata M, Uchida A, Aozasa K, Okamoto M, Kurosawa T, Ochi T, Greene MI, Tsujimoto Y (1995). Moloney murine leukemia virus infection accelerates lymphomagenesis in E mu-bcl-2 transgenic mice. Oncogene.

[27] Baron BW, Anastasi J, Hyjek EM, Bies J, Reddy PL, Dong J, Joseph L, Thirman MJ, Wroblewski K, Wolff L, Baron JM (2012). PIM1 gene cooperates with human BCL6 gene to promote the development of lymphomas. Proc Natl Acad Sci U S A.

[28] Blyth K, Terry A, Mackay N, Vaillant F, Bell M, Cameron ER, Neil JC, Stewart M (2001). Runx2: a novel oncogenic effector revealed by in vivo complementation and retroviral tagging. Oncogene.

[29] Feldman BJ, Reid TR, Cleary ML (1997). Pim1 cooperates with E2a-Pbx1 to facilitate the progression of thymic lymphomas in transgenic mice. Oncogene.

[30] Jonkers J, Korswagen HC, Acton D, Breuer M, Berns A (1997). Activation of a novel proto-oncogene, Frat1, contributes to progression of mouse T-cell lymphomas. EMBO J.

[31] An N, Kraft AS, Kang Y (2013). Abnormal hematopoietic phenotypes in Pim kinase triple knockout mice. J Hematol Oncol.

[32] Fox CJ, Hammerman PS, Thompson CB (2005). The Pim kinases control rapamycin-resistant T cell survival and activation. J Exp Med.

[33] Wang Z, Bhattacharya N, Weaver M, Petersen K, Meyer M, Gapter L, Magnuson NS (2001). Pim-1: A serine/threonine kinase with a role in cell survival, proliferation, differentiation and tumorigenesis. J Vet Sci.

[34] Cuypers HT, Selten G, Berns A, van Kessel AH G (1986). Assignment of the human homologue of Pim-1, a mouse gene implicated in leukemogenesis, to the pter-q12 region of chromosome 6. Hum Genet.

[35] Aho TL, Sandholm J, Peltola KJ, Mankonen HP, Lilly M, Koskinen PJ (2004). Pim-1 kinase promotes inactivation of the pro-apoptotic Bad protein by phosphorylating it on the Ser112 gatekeeper site. FEBS Lett.

[36] Macdonald A, Campbell DG, Toth R, McLauchlan H, Hastie CJ, Arthur JS (2006). Pim kinases phosphorylate multiple sites on Bad and promote 14-3-3 binding and dissociation from Bcl-XL. BMC Cell Biol.

[37] Yan B, Zemskova M, Holder S, Chin V, Kraft A, Koskinen PJ, Lilly M (2003). The PIM-2 kinase phosphorylates BAD on serine 112 and reverses BAD-induced cell death. J Biol Chem.

[38] Li YY, Popivanova BK, Nagai Y, Ishikura H, Fujii C, Mukaida N (2006). Pim-3, a proto-oncogene with serine/ threonine kinase activity, is aberrantly expressed in human pancreatic cancer and phosphorylates bad to block bad-mediated apoptosis in human pancreatic cancer cell lines. Cancer Res.

[39] Gu JJ, Wang Z, Reeves R, Magnuson NS (2009). PIM1 phosphorylates and negatively regulates ASK1-mediated apoptosis. Oncogene.

[40] Hogan C, Hutchison C, Marcar L, Milne D, Saville M, Goodlad J, Kernohan N, Meek D (2008). Elevated levels of oncogenic protein kinase Pim-1 induce the p53 pathway in cultured cells and correlate with increased Mdm2 in mantle cell lymphoma. J Biol Chem.

[41] Zhou BP, Liao Y, Xia W, Zou Y, Spohn B, Hung MC (2001). HER-2/neu induces p53 ubiquitination via Akt-mediated MDM2 phosphorylation. Nat Cell Biol.

[42] Mayo LD, Donner DB (2001). A phosphatidylinositol 3-kinase/Akt pathway promotes translocation of Mdm2 from the cytoplasm to the nucleus. Proc Natl Acad Sci U S A.

[43] Ashcroft M, Ludwig RL, Woods DB, Copeland TD, Weber HO, MacRae EJ, Vousden KH (2002). Phosphorylation of HDM2 by Akt. Oncogene.

[44] Gottlieb TM, Leal JF, Seger R, Taya Y, Oren M (2002). Cross-talk between Akt, p53 and Mdm2: possible implications for the regulation of apoptosis. Oncogene.

[45] Jackson MW, Patt LE, LaRusch GA, Donner DB, Stark GR, Mayo LD (2006). Hdm2 nuclear export, regulated by insulin-like growth factor-I/MAPK/p90Rsk signaling, mediates the transformation of human cells. J Biol Chem.

[46] Weber HO, Ludwig RL, Morrison D, Kotlyarov A, Gaestel M, Vousden KH (2005). HDM2 phosphorylation by MAPKAP kinase 2. Oncogene.

[47] Stott FJ, Bates S, James MC, McConnell BB, Starborg M, Brookes S, Palmero I, Ryan K, Hara E, Vousden KH, Peters G (1998). The alternative product from the human CDKN2A locus, p14(ARF), participates in a regulatory feedback loop with p53 and MDM2. EMBO J.

[48] Llanos S, Clark PA, Rowe J, Peters G (2001). Stabilization of p53 by p14ARF without relocation of MDM2 to the nucleolus. Nat Cell Biol.

[49] Hammerman PS, Fox CJ, Cinalli RM, Xu A, Wagner JD, Lindsten T, Thompson CB (2004). Lymphocyte transformation by Pim-2 is dependent on nuclear factor-kappaB activation. Cancer Res.

[50] Nihira K, Ando Y, Yamaguchi T, Kagami Y, Miki Y, Yoshida K (2010). Pim-1 controls NF-kappaB signalling by stabilizing RelA/p65. Cell Death Differ.

[51] Wang Z, Bhattacharya N, Mixter PF, Wei W, Sedivy J, Magnuson NS (2002). Phosphorylation of the cell cycle inhibitor p21Cip1/WAF1 by Pim-1 kinase. Biochim Biophys Acta.

[52] Wang Z, Zhang Y, Gu JJ, Davitt C, Reeves R, Magnuson NS (2010). Pim-2 phosphorylation of p21(Cip1/WAF1) enhances its stability and inhibits cell proliferation in HCT116 cells. Int J Biochem Cell Biol.

[53] Morishita D, Katayama R, Sekimizu K, Tsuruo T, Fujita N (2008). Pim kinases promote cell cycle progression by phosphorylating and down-regulating p27Kip1 at the transcriptional and posttranscriptional levels. Cancer Res.

[54] Xia W, Chen JS, Zhou X, Sun PR, Lee DF, Liao Y, Zhou BP, Hung MC (2004). Phosphorylation/cytoplasmic localization of p21Cip1/WAF1 is associated with HER2/neu overexpression and provides a novel combination predictor for poor prognosis in breast cancer patients. Clin Cancer Res.

[55] Zhou BP, Liao Y, Xia W, Spohn B, Lee MH, Hung MC (2001). Cytoplasmic localization of p21Cip1/WAF1 by Akt-induced phosphorylation in HER-2/neu-overexpressing cells. Nat Cell Biol.

[56] Mumenthaler SM, Ng PY, Hodge A, Bearss D, Berk G, Kanekal S, Redkar S, Taverna P, Agus DB, Jain A (2009). Pharmacologic inhibition of Pim kinases alters prostate cancer cell growth and resensitizes chemoresistant cells to taxanes. Mol Cancer Ther.

[57] Mochizuki T, Kitanaka C, Noguchi K, Muramatsu T, Asai A, Kuchino Y (1999). Physical and functional interactions between Pim-1 kinase and Cdc25A phosphatase. Implications for the Pim-1-mediated activation of the c-Myc signaling pathway. J Biol Chem.

[58] Bachmann M, Hennemann H, Xing PX, Hoffmann I, Moroy T (2004). The oncogenic ser- ine/threonine kinase Pim-1 phosphorylates and inhibits the activity of Cdc25C-associated kinase 1 (C-TAK1): a novel role for Pim-1 at the G2/M cell cycle checkpoint. J Biol Chem.

[59] Bhattacharya N, Wang Z, Davitt C, McKenzie IF, Xing PX, Magnuson NS (2002). Pim-1 associates with protein complexes necessary for mitosis. Chromosoma.

[60] Yip-Schneider MT, Horie M, Broxmeyer HE (1995). Transcriptional induction of pim-1 protein kinase gene expression by interferon gamma and posttranscriptional effects on costimulation with steel factor. Blood.

[61] Chen XP, Losman JA, Cowan S, Donahue E, Fay S, Vuong BQ, Nawijn MC, Capece D, Cohan VL, Rothman P (2002). Pim serine/threonine kinases regulate the stability of Socs-1 protein. Proc Natl Acad Sci U S A.

[62] Peltola KJ, Paukku K, Aho TL, Ruuska M, Silvennoinen O, Koskinen PJ (2004). Pim-1 kinase inhibits STAT5-dependent transcription via its interactions with SOCS1 and SOCS3. Blood.

[63] Chen JL, Limnander A, Rothman PB (2008). Pim-1 and Pim-2 kinases are required for efficient pre-B-cell transformation by v-Abl oncogene. Blood.

[64] Berns A, Mikkers H, Krimpenfort P, Allen J, Scheijen B, Jonkers J (1999). Identification and characterization of collaborating oncogenes in compound mutant mice. Cancer Res.

[65] Shirogane T, Fukada T, Muller JM, Shima DT, Hibi M, Hirano T (1999). Synergistic roles for Pim-1 and c-Myc in STAT3-mediated cell cycle progression and antiapoptosis. Immunity.

[66] Zhang Y, Wang Z, Li X, Magnuson NS (2008). Pim kinase‐dependent inhibition of c‐Myc degradation. Oncogene.

[67] Zippo A, De Robertis A, Serafini R, Oliviero S (2007). PIM1-dependent phosphorylation of histone H3 at serine 10 is required for MYC-dependent transcriptional activation and oncogenic transformation. Nat Cell Biol.

[68] Winter S, Simboeck E, Fischle W, Zupkovitz G, Dohnal I, Mechtler K, Ammerer G, Seiser C (2008). 14‐3‐3 proteins recognize a histone code at histone H3 and are required for transcriptional activation. EMBO J.

[69] Zippo A, Serafini R, Rocchigiani M, Pennacchini S, Krepelova A, Oliviero S (2009). Histone crosstalk between H3S10ph and H4K16ac generates a histone code that mediates transcription elongation. Cell.

[70] Nakayama KI, Nakayama K (2006). Ubiquitin ligases: cell-cycle control and cancer. Nat Rev Cancer.

[71] Matsumoto A, Onoyama I, Nakayama KI (2006). Expression of mouse Fbxw7 isoforms is regulated in a cell cycle- or p53-dependent manner. Biochem Biophys Res Commun.

[72] Yoshida GJ, Saya H (2014). Inversed relationship between CD44 variant and c-Myc due to oxidative stress-induced canonical Wnt activation. Biochem Biophys Res Commun.

[73] Reavie L, Della Gatta G, Crusio K, Aranda-Orgilles B, Buckley SM, Thompson B, Lee E, Gao J, Bredemeyer AL, Helmink BA, Zavadil J, Sleckman BP, Palomero T, Ferrando A, Aifantis I (2010). Regulation of hematopoietic stem cell differentiation by a single ubiquitin ligase-substrate complex. Nat Immunol.

[74] Reavie L, Buckley SM, Loizou E, Takeishi S, Aranda-Orgilles B, Ndiaye-Lobry D, Abdel-Wahab O, Ibrahim S, Nakayama KI, Aifantis I (2013). Regulation of c-Myc ubiquitination controls chronic myelogenous leukemia initiation and progression. Cancer Cell.

[75] Kwak EL, Moberg KH, Wahrer DC, Quinn JE, Gilmore PM, Graham CA, Hariharan IK, Harkin DP, Haber DA, Bell DW (2005). Infrequent mutations of Archipelago (hAGO, hCDC4, Fbw7) in primary ovarian cancer. Gynecol Oncol.

[76] Strohmaier H, Spruck CH, Kaiser P, Won KA, Sangfelt O, Reed SI (2001). Human F-box protein hCdc4 targets cyclin E for proteolysis and is mutated in a breast cancer cell line. Nature.

[77] Spruck CH, Strohmaier H, Sangfelt O, Muller HM, Hubalek M, Muller-Holzner E, Marth C, Widschwendter M, Reed SI (2002). hCDC4 gene mutations in endometrial cancer. Cancer Res.

[78] Rajagopalan H, Jallepalli PV, Rago C, Velculescu VE, Kinzler KW, Vogelstein B, Lengauer C (2004). Inactivation of hCDC4 can cause chromosomal instability. Nature.

[79] Siu KT, Xu Y, Swartz KL, Bhattacharyya M, Gurbuxani S, Hua Y, Minella AC (2014). Chromosome instability underlies hematopoietic stem cell dysfunction and lymphoid neoplasia associated with impaired Fbw7- mediated cyclin E regulation. Mol Cell Biol.

[80] Chang TC, Yu D, Lee YS, Wentzel EA, Arking DE, West KM, Dang CV, Thomas-Tikhonenko A, Mendell JT (2008). Widespread microRNA repression by Myc contributes to tumorigenesis. Nat Genet.

[81] Liu L, Wang S, Chen R, Wu Y, Zhang B, Huang S, Zhang J, Xiao F, Wang M, Liang Y (2012). Myc induced miR-144/451 contributes to the acquired imatinib resistance in chronic myelogenous leukemia cell K562. Biochem Biophys Res Commun.

[82] Hammerman PS, Fox CJ, Brinbaum MJ, Thompson CB (2005). Pim and Akt oncogenes are independent regulators of hematopoietic cell growth and survival. Blood.

[83] Schatz JH, Oricchio E, Wolfe AL, Jiang M, Linkov I, Maragulia J, Shi W, Zhang Z, Rajasekhar VK, Pagano NC, Porco JA, Teruya-Feldstein J, Rosen N, Zelenetz AD, Pelletier J, Wendel HG (2011). Targeting cap-dependent translation blocks converging survival signals by AKT and PIM kinases in lymphoma. J Exp Med.

[84] Cen B, Xiong Y, Song JH, Mahajan S, DuPont R, McEachern K, DeAngelo DJ, Cortes JE, Minden MD, Ebens A, Mims A, LaRue AC, Kraft AS (2014). The Pim-1 Protein Kinase Is an Important Regulator of MET Receptor Tyrosine Kinase Levels and Signaling. Mol Cell Biol.

[85] Kucia M, Jankowski K, Reca R, Wysoczynski M, Bandura L, Allendorf D, Zhang J, Ratajczak J, Ratajczak M (2004). CXCR4–SDF-1 signaling, locomotion, chemotaxis and adhesion. J Mol Histol.

[86] Lapidot T, Dar A, Kollet O (2005). How do stem cells find their way home?. Blood.

[87] Busillo JM, Benovic JL (2007). Regulation of CXCR4 signaling. Biochim Biophys Acta.

[88] Boudot A, Kerdivel G, Lecomte S, Flouriot G, Desille M, Godey F, Leveque J, Tas P, Le Dréan Y, Pakdel F (2014). COUP-TFI modifies CXCL12 and CXCR4 expression by activating EGF signaling and stimulates breast cancer cell migration. BMC Cancer.

[89] Matteucci E, Locati M, Desiderio MA (2005). Hepatocyte growth factor enhances CXCR4 expression favoring breast cancer cell invasiveness. Exp Cell Res.

[90] Chetram MA, Don-Salu-Hewage AS, Hinton CV (2011). ROS enhances CXCR4-mediated functions through inactivation of PTEN in prostate cancer cells. Biochem Biophys Res Commun.

[91] Matsusaka S, Tohyama Y, He J, Shi Y, Hazama R, Kadono T, Kurihara R, Tohyama K, Yamamura H (2005). Protein-tyrosine kinase, Syk, is required for CXCL12-induced polarization of B cells. Biochem Biophys Res Commun.

[92] Croker AK, Allan AL (2008). Cancer stem cells: implications for the progression and treatment of metastatic disease. J Cell Mol Med.

[93] Grundler R, Brault L, Gasser C, Bullock AN, Dechow T, Woetzel S, Pogacic V, Villa A, Ehret S, Berridge G, Spoo A, Dierks C, Biondi A, Knapp S, Duyster J, Schwaller J (2009). Dissection of PIM serine/threonine kinases in FLT3-ITD- induced leukemogenesis reveals PIM1 as regulator of CXCL12-CXCR4-mediated homing and migration. J Exp Med.

[94] Decker S, Finter J, Forde AJ, Kissel S, Schwaller J, Mack TS, Kuhn A, Gray N, Follo M, Jumaa H, Burger M, Zirlik K, Pfeifer D, Miduturu CV, Eibel H, Veelken H, Dierks C (2014). PIM kinases are essential for chronic lymphocytic leukemia cell survival (PIM2/3) and CXCR4-mediated microenvironmental interactions (PIM1). Mol Cancer Ther.

[95] Chen W, Kumar AR, Hudson WA, Li Q, Wu B, Staggs RA, Lund EA, Sam TN, Kersey JH (2008). Gene dosage and critical target cells. Cancer Cell.

[96] Mizuki M, Schwable J, Steur C, Choudhary C, Agrawal S, Sargin B, Steffen B, Matsumura I, Kanakura Y, Böhmer FD, Müller-Tidow C, Berdel WE, Serve H (2003). Suppression of myeloid transcription factors and induction of STAT response genes by AML-specific Flt3 mutations. Blood.

[97] Hu YL, Passegue E, Fong S, Largman C, Lawrence HJ (2007). Evidence that the Pim1 kinase gene is a direct target of HOXA9. Blood.

[98] Vu HA, Xinh PT, Kano Y, Tokunaga K, Sato Y (2009). The juxtamembrane domain in ETV6/FLT3 is critical for PIM-1 up-regulation and cell proliferation. Biochem Biophys Res Commun.

[99] Gomez-Abad C, Pisonero H, Blanco-Aparicio C, Roncador G, González-Menchén A, Martinez-Climent JA, Mata E, Rodríguez ME, Muñoz-González G, Sánchez-Beato M, Leal JF, Bischoff JR, Piris MA (2011). PIM2 inhibition as a rational therapeutic approach in B-cell lymphoma. Blood.

[100] Brault L, Menter T, Obermann EC, Knapp S, Thommen S, Schwaller J, Tzankov A (2012). PIM kinases are progression markers and emerging therapeutic targets in diffuse large B-cell lymphoma. Br J Cancer.

[101] Hsi ED, Jung SH, Lai R, Johnson JL, Cook JR, Jones D, Devos S, Cheson BD, Damon LE, Said J (2008). Ki67 and PIM1 expression predict outcome in mantle cell lymphoma treated with high dose therapy, stem cell transplantation and rituximab: a Cancer and Leukemia Group B 59909 correlative science study. Leuk Lymphoma.

[102] Pasqualucci L, Neumeister P, Goossens T, Nanjangud G, Chaganti RS, Küppers R, Dalla-Favera R (2001). Hypermutation of multiple proto-oncogenes in B-cell diffuse large-cell lymphomas. Nature.

[103] Claudio JO, Masih-Khan E, Tang H, Gonçalves J, Voralia M, Li ZH, Nadeem V, Cukerman E, Francisco-Pabalan O, Liew CC, Woodgett JR, Stewart AK (2002). A molecular compendium of genes expressed in multiple myeloma. Blood.

[104] Pogacic V, Bullock AN, Fedorov O, Filippakopoulos P, Gasser C, Biondi A, Meyer-Monard S, Knapp S, Schwaller J (2007). Structural analysis identifies imidazo[1,2-b]pyridazines as PIM kinase inhibitors with in vitro antileukemic activity. Cancer Res.

[105] Blanco-Aparicio C, Collazo AM, Oyarzabal J, Leal JF, Albarán MI, Lima FR, Pequeño B, Ajenjo N, Becerra M, Alfonso P, Reymundo MI, Palacios I, Mateos G, Quiñones H, Corrionero A, Carnero A, Pevarello P, Lopez AR, Fominaya J, Pastor J, Bischoff JR (2011). Pim 1 kinase inhibitor ETP-45299 suppresses cellular proliferation and synergizes with PI3K inhibition. Cancer Lett.

[106] Garcia PD, Langowski JL, Wang Y, Chen M, Castillo J, Fanton C, Ison M, Zavorotinskaya T, Dai Y, Lu J, Niu XH, Basham S, Chan J, Yu J, Doyle M, Feucht P, Warne R, Narberes J, Tsang T, Fritsch C, Kauffmann A, Pfister E, Drueckes P, Trappe J, Wilson C, Han W, Lan J, Nishiguchi G, Lindvall M, Bellamacina C, Aycinena JA, Zang R, Holash J, Burger MT (2014). Pan-PIM kinase inhibition provides a novel therapy for treating hematologic cancers. Clin Cancer Res.

[107] Chen LS, Redkar S, Bearss D, Wierda WG, Gandhi V (2009). Pim kinase inhibitor, SGI-1776, induces apoptosis in chronic lymphocytic leukemia cells. Blood.

[108] Chen LS, Redkar S, Taverna P, Cortes JE, Gandhi V (2011). Mechanisms of cytotoxicity to Pim kinase inhibitor, SGI-1776, in acute myeloid leukemia. Blood.

[109] Yang Q, Chen LS, Neelapu SS, Miranda RN, Medeiros LJ, Gandhi V (2012). Transcription and translation are primary targets of Pim kinase inhibitor SGI-1776 in mantle cell lymphoma. Blood.

[110] Hospital MA, Green AS, Lacombe C, Mayeux P, Bouscary D, Tamburini J (2012). The FLT3 and Pim kinases inhibitor SGI-1776 preferentially target FLT3-ITD AML cells. Blood.

[111] Fischer KM, Cottage CT, Konstandin MH, Völkers M, Khan M, Sussman MA (2011). Pim-1 kinase inhibits pathological injury by promoting cardioprotective signaling. J Mol Cell Cardiol.

[112] Quijada P, Toko H, Fischer KM, Bailey B, Reilly P, Hunt KD, Gude NA, Avitabile D, Sussman MA (2012). Preservation of myocardial structure is enhanced by pim-1 engineering of bone marrow cells. Circ Res.

[113] Lin YW, Beharry ZM, Hill EG, Song JH, Wang W, Xia Z, Zhang Z, Aplan PD, Aster JC, Smith CD, Kraft AS (2010). A small molecule inhibitor of Pim protein kinases blocks the growth of precursor T-cell lymphoblastic leukemia/lymphoma. Blood.

[114] Beharry Z, Zemskova M, Mahajan S, Zhang F, Ma J, Xia Z, Lilly M, Smith CD, Kraft AS (2009). Novel benzylidene-thiazolidine-2,4-diones inhibit Pim protein kinase activity and induce cell cycle arrest in leukemia and prostate cancer cells. Mol Cancer Ther.

[115] Dakin LA, Block MH, Chen H, Code E, Dowling JE, Feng X, Ferguson AD, Green I, Hird AW, Howard T, Keeton EK, Lamb ML, Lyne PD, Pollard H, Read J, Wu AJ, Zhang T, Zheng X (2012). Discovery of novel benzylidene-1,3-thiazolidine-2,4-diones as potent and selective inhibitors of the PIM-1, PIM-2, and PIM-3 protein kinases. Bioorg Med Chem Lett.

[116] Meja K, Stengel C, Sellar R, Huszar D, Davies BR, Gale RE, Linch DC, Khwaja A (2014). PIM and AKT kinase inhibitors show synergistic cytotoxicity in acute myeloid leukaemia that is associated with convergence on mTOR and MCL1 pathways. Br J Haematol.

[117] Brzózka K, Windak R, Guratowska M, Krawczyńska K, Kłosowska-Wardęga A, Zurawska M, Trębacz E, Sabiniarz A, Czardybon W, Chołody M, Horvath R, Szamborska-Gbur A, Prymula K, Milik M, Kowalczyk P, Rzymski T, Beuzen N (2012). Preclinical development of a Pim kinase inhibitor for cancer treatment. [abstract]. Cancer Res.

[118] Keeton EK, McEachern K, Dillman KS, Palakurthi S, Cao Y, Grondine MR, Kaur S, Wang S, Chen Y, Wu A, Shen M, Gibbons FD, Lamb ML, Zheng X, Stone RM, Deangelo DJ, Platanias LC, Dakin LA, Chen H, Lyne PD, Huszar D (2014). AZD1208, a potent and selective pan-Pim kinase inhibitor, demonstrates efficacy in preclinical models of acute myeloid leukemia. Blood.

[119] Fathi AT, Arowojolu O, Swinnen I, Sato T, Rajkhowa T, Small D, Marmsater F, Robinson JE, Gross SD, Martinson M, Allen S, Kallan NC, Levis M (2012). A potential therapeutic target for FLT3-ITD AML: PIM1 kinase. Leuk Res.

