# Supplementary figures and images for: Pim kinases in hematological malignancies: where are we now and where are we going?
Source: J Hematol Oncol. 2014 Dec 10;7:95. doi: 10.1186/s13045-014-0095-z (PMC4266197; doi:10.1186/s13045-014-0095-z)

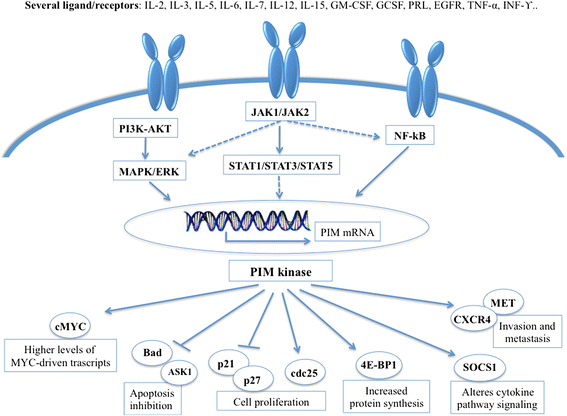

Supplement: Supplementary file 1 — Authors’ original file for figure 1 [file 13045_2014_95_MOESM1_ESM.gif]
