# Supplementary material for: Pim kinases in hematological malignancies: where are we now and where are we going?
Source: J Hematol Oncol. 2014 Dec 10;7:95. doi: 10.1186/s13045-014-0095-z (PMC4266197; doi:10.1186/s13045-014-0095-z)
Supplement: Supplementary file 2 — Authors’ original file for figure 2 [file 13045_2014_95_MOESM2_ESM.doc]

**Table 1**. Novel Pim-Inhibitors in hematologic malignancies.

| **Compound** | **Class** | **PIM inhibition**  **Selectivity** | **Development** | **Disease** |
| --- | --- | --- | --- | --- |
| **SGI-­1776** | imadizaopyridazine | IC50: 7 nM PIM1, 363 nM PIM2, 69 nM PIM3, 44 nM FLT-3 and 34 nM Haspin [107] | Failure in phase I clinical trials by cardiotoxicity | Non-Hodgkin lymphoma |
| **SMI4a** | benzylidene-thiazolidene-2,4-dione | IC50: 21 nM PIM1, 100 nM PIM2. [113]  Selective vs. 56 kinases. [114] | Preclinical | Acute myeloid leukemia |
| **LGB321** | 3-(S)-amino-piperidine pyridyl carboxamide | IC50: 0.001 nM PIM1, 0.0021 nM PIM2, and 0.0008 nM PIM3. [106] | Recruiting patients for clinical trials | Multiple myeloma |
| **AZD1897** |  | IC50: 3 nM PIM1,2 and 3. [115] | Preclinical | Acute myeloid leukemia |
| **SEL24-B58** | Benzoimidazol | IC50: 31 nM PIM1, 154 nM PIM2, 152 nM PIM3. Selective in a panel of 299 kinases with the exception of haspin, HIPK and CLK kinases. [117] | Preclinical | Leukemic monocyte lymphoma |
| **AZD1208** | thiazolidene | IC50: Pim-1 0.4 nM, Pim-2 5.0 nM and Pim-3 1.9 nM. [115] | Recruiting patients for clinical trials | Acute myeloid leukemia |
